# A Framework for Criteria-Based Selection and Processing of Fast Healthcare Interoperability Resources (FHIR) Data for Statistical Analysis: Design and Implementation Study

**DOI:** 10.2196/25645

**Published:** 2021-04-01

**Authors:** Julian Gruendner, Christian Gulden, Marvin Kampf, Sebastian Mate, Hans-Ulrich Prokosch, Jakob Zierk

**Affiliations:** 1 Chair of Medical Informatics Department of Medical Informatics, Biometrics and Epidemiology Friedrich-Alexander University Erlangen-Nürnberg Erlangen-Tennenlohe Germany; 2 Medical Center for Information and Communication Technology University Hospital Erlangen Erlangen Germany; 3 Department of Pediatrics and Adolescent Medicine University Hospital Erlangen Erlangen Germany

**Keywords:** data analysis, data science, data standardization, digital medical information, eHealth, Fast Healthcare Interoperability Resources, data harmonization, medical information, patient privacy, data repositories, HL7 FHIR

## Abstract

**Background:**

The harmonization and standardization of digital medical information for research purposes is a challenging and ongoing collaborative effort. Current research data repositories typically require extensive efforts in harmonizing and transforming original clinical data. The Fast Healthcare Interoperability Resources (FHIR) format was designed primarily to represent clinical processes; therefore, it closely resembles the clinical data model and is more widely available across modern electronic health records. However, no common standardized data format is directly suitable for statistical analyses, and data need to be preprocessed before statistical analysis.

**Objective:**

This study aimed to elucidate how FHIR data can be queried directly with a preprocessing service and be used for statistical analyses.

**Methods:**

We propose that the binary JavaScript Object Notation format of the PostgreSQL (PSQL) open source database is suitable for not only storing FHIR data, but also extending it with preprocessing and filtering services, which directly transform data stored in FHIR format into prepared data subsets for statistical analysis. We specified an interface for this preprocessor, implemented and deployed it at University Hospital Erlangen-Nürnberg, generated 3 sample data sets, and analyzed the available data.

**Results:**

We imported real-world patient data from 2016 to 2018 into a standard PSQL database, generating a dataset of approximately 35.5 million FHIR resources, including “Patient,” “Encounter,” “Condition” (diagnoses specified using International Classification of Diseases codes), “Procedure,” and “Observation” (laboratory test results). We then integrated the developed preprocessing service with the PSQL database and the locally installed web-based KETOS analysis platform. Advanced statistical analyses were feasible using the developed framework using 3 clinically relevant scenarios (data-driven establishment of hemoglobin reference intervals, assessment of anemia prevalence in patients with cancer, and investigation of the adverse effects of drugs).

**Conclusions:**

This study shows how the standard open source database PSQL can be used to store FHIR data and be integrated with a specifically developed preprocessing and analysis framework. This enables dataset generation with advanced medical criteria and the integration of subsequent statistical analysis. The web-based preprocessing service can be deployed locally at the hospital level, protecting patients’ privacy while being integrated with existing open source data analysis tools currently being developed across Germany.

## Introduction

### Background

With an increase in digitalization in the medical sciences, the efforts to harmonize and standardize clinical data have increased. In particular, transformation of data sets into a common format has received increasing attention to render the data queryable and allow for standardized model building. Two research data repositories with appropriate analysis environments, which have been used extensively and received increasing support, are the OHDSI OMOP common data model [1], which has been designed to facilitate observational research, and Informatics for Integrating Biology and the Bedside (i2b2) [2], which focuses on the integration of different types of data into one clinical repository. Both OHDSI OMOP and i2b2 aim to transform clinical data to a standardized format and vocabulary and are appropriate for research and further analysis. However, importing of data requires the complex implementation of extract, transform, load (ETL) processes [3].

Conversely, the Fast Healthcare Interoperability Resource (FHIR) standard was developed to address the limitations of the previously developed HL7 versions 2 and 3 clinical care document standards; therefore, it is focused on modeling the actual clinical environment as closely as possible. Furthermore, its lightweight nature and direct use of common data formats (ie, JSON and XML) facilitate integration with lightweight webservices. FHIR is now available in its first release with normative resource specifications since version 4.0.0 in 2019 [4], suggesting further maturation of this standard. Large companies including Google, Microsoft, and Apple have adopted FHIR for their medical informatics–related products [5-7]. Moreover, many health system providers are now striving to support or are already supporting the FHIR standard [8], thus potentially facilitating the integration of new solutions into clinical routine, as complex conversions into standards, such as OMOP and i2b2, can be avoided when solutions are deployed within hospitals.

The German Medical Informatics Initiative (MI-I) [9] has recently funded 4 consortia across Germany to investigate how heterogenous clinical data can be integrated into clinical data repositories. One of the objectives of the MI-I is to establish data integration centers (DICs) as the base for cross-hospital and cross-consortia communication. These DICs would provide different services including data integration, data harmonization, standardized data repositories, consent management, and ID management [10-13]. The MI-I has adopted FHIR as the preferred format for inter-consortia communication [14]. All 34 hospitals that are currently part of the MI-I will have a FHIR store available in one form or another and have committed to making their hospital data available in the FHIR format.

The current state of the analysis of FHIR formatted data remains unclear. One drawback of FHIR is that formats such as JSON and XML are not necessarily suitable for further analysis if data are stored in these formats and not processed further. The FHIR standard itself contains an extensive specification for API search operations [15], which, in turn, have their limitations [16]. Specifically, it is not directly possible to express queries with interdata dependencies and necessary computations. Furthermore, searching for resources on the basis of inclusion and exclusion criteria is not possible if they are based on another resource that is not referenced directly but rather indirectly via another intermediary resource. To account for these limitations and to support more complex statistical analysis, a query engine is needed, which should be accessible to researchers without the knowledge of SQL or database query generation and optimization.

Over the years, different FHIR databases have been developed to address the limitations of the FHIR search specification. The blaze FHIR store not only implements the FHIR interface but also introduces the possibility of using clinical quality language to further improve the standard FHIR search and filter possibilities [17]. This platform focuses on feasibility queries and data exports. Another alternative to enhance the availability of FHIR data in an easily accessible manner is to use the PostgreSQL (PSQL) database [18] owing to its innate capability to store, index, and query JSON as binary JSON (jsonb). The fhirbase [19] FHIR database uses PSQL and implements a SQL query interface, which allows a user to query FHIR resources using the SQL syntax. Neither of these solutions currently offer a user-friendly method for a researcher to filter and select data for further statistical analysis, which does not require a strong technical background.

### Aim

This study aimed to investigate how a data preprocessing service can be built directly on top of FHIR data stored in a standard PSQL database to enable large data filter queries to generate data sets for statistical analysis. To investigate the requirements for filtering and subset generation, we identified different sample medical data science scenarios. Based on the scenarios’ requirements, we defined a data preprocessor, which generates data subsets on the basis of the inclusion and exclusion criteria of other FHIR resources. This data preprocessor was developed to satisfy the demand for investigating subsets of particular FHIR observations and combine them with basic patient data. In this study, which was approved by the institutional review board of the University Hospital Erlangen-Nürnberg (reference# 254_19 Bc), we integrated the developed preprocessing service with a real-world FHIR data set from our hospital, which—at the time of writing—contained approximately 35.5 million FHIR resources. Using this data set, we implemented three different sample medical questions to investigate the capabilities of the implemented web-based preprocessing service. Furthermore, we integrated the service into the locally deployed web-based analysis platform KETOS [20], which enables data retrieval and analysis using Jupyter Notebooks [21] within the hospital, thus respecting a patients’ privacy and allowing the data custodians to have ownership of the data.

## Methods

### Requirements for a Preprocessing Service

Analysis of large data sets using various statistical methods requires the data to be standardized and harmonized. Additionally, the data set needs to be transformed into a format suitable for further analysis (ie, a “flat structure”). A common approach to this end is to select a subset of data and then convert the selected subset into a simple tabular format. To determine which type of filters are commonly required, we referred to our ongoing multicenter medical research study, wherein we analyzed laboratory findings that are filtered in accordance with the patients’ clinical criteria, including patients’ diagnoses, clinical procedures, and the results of other laboratory analyses. Specific time criteria (eg, time intervals) are defined for each criterion. This analysis allowed us to identify the following requirements: the ability to select a subset of resources and exclude data from these resources on the basis of their relationship with other resources as inclusion and exclusion criteria. Further, these resources would have to be preprocessed on premises and integrated with the existing DIC infrastructure, so that the analysis could be performed within the hospital on pseudonymized data to adhere to patient privacy and data security regulations in Germany.

### Integration With the Existing Infrastructure: the German DIC

**Figure 1:**
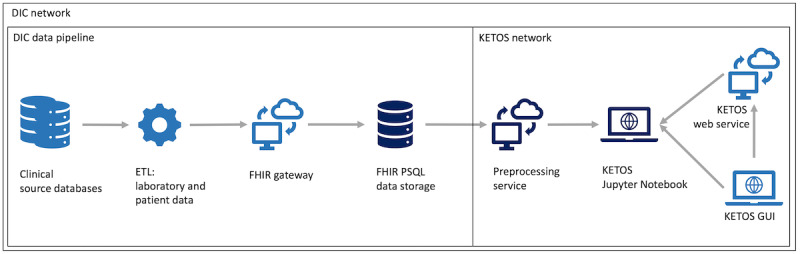
Integration with the infrastructure of the data integration center: data storage, preprocessing, and analysis environment. DIC: data integration center, ETL: extract transform and load, FHIR: Fast Healthcare Interoperability Resources, PSQL: PostgreSQL.

We propose that the web-based nature and the reliance on a standard PSQL database with only one table ensures the easy integration of this system into existing infrastructure. Figure 1 shows some components of the DIC infrastructure and some of the analysis tools currently being developed in Germany. The DIC, as currently deployed across 10 German University Hospitals, includes ETL jobs to convert existing data into the FHIR format; moreover, it has a FHIR gateway component, which accepts FHIR resources and loads them into a FHIR PSQL database. This PSQL database, which is the focus of this study, is a standard PSQL database that contains a single table with the following columns: id, fhir_id, type, and data. The data column contains the respective FHIR resource in jsonb format, allowing one to query each element of the JSON stored data directly, while providing complete functionality of a PSQL relational database, like JOINS, timestamp conversion, and LIKE pattern searches. Therefore, a preprocessing service built on this data structure could be run within any hospital as long as the FHIR gateway and the FHIR PSQL database are installed. The entire infrastructure is available in the form of Docker containers and can be easily distributed to other sites. The preprocessing service in this study is web-based and hence integrates well with other web-based platforms for further analysis, such as the KETOS platform for statistical analysis.

### The Data Set

The FHIR PSQL database, which we connected our preprocessing service to, contains data on 170,389 patients and 323,779 encounters over 3 years from 2016 to 2018. Among these patients, 88,473 were female, 81,914 were male, and 2 were of an unknown or unspecified gender.

The data sources included the hospital’s standardized billing data, which each German hospital is legally required to provide, and laboratory data from a local data warehouse. These data had been harmonized, and laboratory data were mapped to the LOINC vocabulary; diagnoses to International Classification of Diseases, Tenth Revision codes; and procedures to OPS codes. Further, the local DIC pseudonymized the data and harmonized the laboratory units of measurement. The final data set derived from the process included 31,697,035 FHIR Observations, of which 31,686,060 were laboratory findings, 1,740,632 were International Classification of Diseases, Tenth Revision–coded FHIR Conditions, 1,637,573 were FHIR Procedures, 132 were FHIR Medications, and 10,348 were FHIR MedicationSatements. After preprocessing this data set, the final subsets were obtained.

### Specification of the Filter Criteria

**Figure 2:**
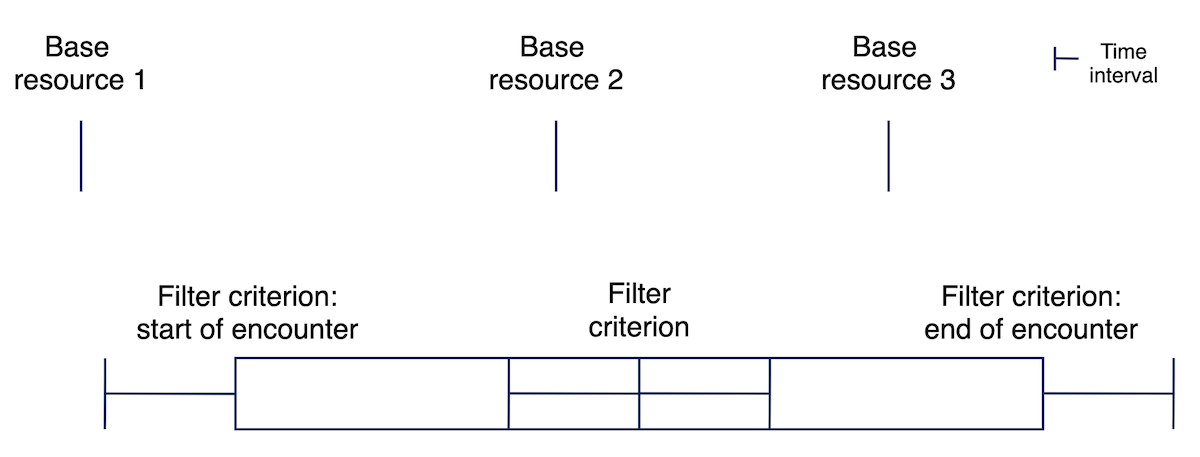
Timeline for filter matching.

Through the aforementioned analysis, we established that the preprocessing service should be able to filter all resources from the initial result set (which we referred to as the “base resources”), either on the basis of inclusion or exclusion filter criteria or a combination of both, where a filter criterion is based on another FHIR resource. The filter would then be applied either if a filter criterion ever matched for a patient or if a criterion matched for a patient within a particular time interval of the resources from the base resource to be filtered. Further, as the time of a laboratory result can often not be directly determined, it was important to determine this period on the basis of the encounter of the filter criterion. If no encounter is available, the laboratory result is filtered on the basis of the criterion along with a time interval. The resulting logic for resource matching based on the time from the base resource is depicted in Figure 2. The figure shows three base resources: 1, 2, and 3. Resource 1 would not be filtered from the result set because it lies outside the specified time interval. Resource 2 would be filtered because it lies within the specified time interval from the filter criterion. Resource 3 would be filtered if the filter criterion has an encounter because it lies within the time interval of the encounter of the filter criterion. However, it would not be filtered if the filter criterion does not have an encounter.

In addition to the possibility of defining the time interval within which a resource must be filtered, filter criteria should be selected on the basis of their respective code (eg, LOINC code 718-7 for hemoglobin). Further, it should be possible to specify a simple value restriction in accordance with standard comparators.

### Data Availability

The source code of the project is available on GitHub [22].

## Results

### Overview of the Findings

We implemented and deployed the preprocessing service that we implemented in this study at the University Hospital Erlangen-Nürnberg. The whole pipeline could be easily deployed on an existing server, as the web-based preprocessing service was packaged as a Docker container [23].

To ensure secure functioning of the preprocessing service, we deployed it on the same server and within the same Docker network as the KETOS analysis environment. The preprocessing service was then only made available within the Docker network on the server and was not accessible outside the KETOS platform. Finally, we applied the preprocessing service to patient data from 2016 to 2018 stored in the local DIC FHIR database (see *The Data Set*). We used the preprocessing service to generate 3 sample data sets and analyses to demonstrate its applicability to clinically relevant research questions. We then analyzed the resulting prepared data sets with the KETOS platform and a Jupyter Notebook (interactive cell-based code development in a web browser).

### Specification of the Preprocessing Service and Data Input

Based on the data analysis and the specification of filter criteria, we described an interface that receives the input parameters in JSON format (Multimedia Appendix 1) and uses the input to generate a PSQL filter query (Multimedia Appendix 2), which is sent to the FHIR PSQL database where the query is executed. This query yields a subset of resources. The preprocessor then generates a feature set using this subset and combines the subset with basic patient data to generate the final feature data set for further statistical analysis, as specified in the feature_set part of the input parameter JSON. The initial filtered resource set and the final feature set are then stored in the preprocessors’ own local database ready to be downloaded for analysis. The preprocessor itself was implemented as a webservice, using the Python Flask-Restful library [24].

### Example 1: Data-Driven Establishment of Reference Intervals

In modern medicine, laboratory tests are an essential tool for health assessment and substantially influence diagnostic and treatment decisions. To support decision making among clinicians, laboratory findings are accompanied by reference intervals, which reflect the range of test results in a population of healthy individuals. Conventionally, reference intervals have been established among specifically recruited healthy individuals (“direct approach”); however, this approach is associated with substantial financial and logistical challenges. Therefore, data-driven approaches (“indirect approaches”) have been developed, which use data from laboratory information systems and statistical analyses to estimate the proportion of samples from healthy individuals in mixed data sets (ie, hospital data sets containing a large fraction of abnormal test results). While indirect approaches can tolerate a high proportion of abnormal findings, their accuracy is limited by the proportion of abnormal samples.

Here, we demonstrate how reference intervals for a very common laboratory test (hemoglobin) can be established using the tools developed in this study and the open source kosmic [25] algorithm, despite a very high proportion of abnormal findings in the analyzed data set (ie, in-patient laboratory findings from a tertiary care center). To reduce the number of abnormal findings, we excluded all patients with cancer diagnoses (defined by ICD codes starting with *C*) and those who received transfusions (defined using OPS codes starting with *8-80*) at any time. Additionally, we excluded all findings from patients with clearly abnormal hemoglobin values (ie, <8.0 g/dL) at any time and those having undergone surgery within 90 days (defined by OPS codes starting with *5-*). We then restricted the data set to a sample of interest (men aged 18-65 years), selected one random finding per patient and used the resulting data set (n=13,721) as input for the kosmic algorithm. This yielded a clinically useful reference interval (13.2-17.2 g/dL), which highlights the potential of the developed framework to handle complex medical data science scenarios.

### Example 2: Anemia in Patients With Cancer

Assessment of differences in laboratory findings among different patient cohorts enhances physicians’ understanding of the pathophysiology of diseases and treatment effects. To assess the feasibility of such analyses using the tools developed in this study, we generated a data set to investigate anemia occurrence (ie, hemoglobin levels below cut-off values defined by the World Health Organization) among adult patients with and those without cancer. We queried the minimum hemoglobin level (defined using LOINC code *718-7*) of patients with and those without cancer (included or excluded using ICD codes starting with *C*) and determined the number of adult patients below anemia-defining thresholds (13 g/dL for men and 12 g/dL for women). In total, this resulted in a data set with 686,472 hemoglobin test results from 9075 men and 9035 women with cancer and 45,766 men and 53,777 women without cancer. We observed a substantially larger proportion of men and women with anemia among patients with cancer (n=6316, 69.6% and n=5674, 62.8%, respectively) than among those without cancer (n=16,247, 35.5% and n=22,586, 42.0%, respectively) (*P*<.001, Fisher exact test). These findings indicate a high prevalence of anemia, a condition associated with substantial morbidity and mortality, in cancer (ie, the second most common cause of death worldwide) and the suitability of the tools developed in this study for such analyses.

### Example 3: Adverse Effects of Drugs

Adverse effects of drugs are a major contributor to patient morbidity and mortality among in-hospital patients and outpatients, and a substantial proportion of drugs’ adverse effects influence laboratory findings. Here, we used the framework developed in this study to generate a data set to investigate changes in patients’ potassium levels during treatment with an important anti-infective drug (liposomal amphotericin B, a potent and essential antifungal agent that decreases potassium levels in some patients). We selected potassium levels (defined using LOINC code *2823-3*) in patients who received liposomal amphotericin B (defined using OPS codes starting with *6-002.q*) within 7 days (study group: 107 patients and 4568 potassium test results) and potassium levels in patients who received liposomal amphotericin B at any time but not within 7 days (control group: 145 patients and 5581 potassium test results). This example shows that this framework can be used to generate a data set to investigate the adverse effects of drugs. Although potassium levels did not significantly differ between both groups in this data set (*P*=.12), they were lower in the study group (3.4 mM) than in the control group (3.5 mM), demonstrating the ability of this framework to investigate the adverse effects of drugs.

## Discussion

### Principal Findings

Direct retrieval of data stored using FHIR resources for further statistical analysis is an important step to bridge the gap between the acquisition of medical data and clinically relevant research. To comply with patient privacy and data security regulations, it is important to establish tools that can be directly deployed within the hospital infrastructure, so that the data remain within the institutions’ network and control. The preprocessor we developed satisfies these concerns and relies on open source tools that can be easily distributed across hospitals to improve future research. Further, since this preprocessor relies on FHIR resources, extra ETL jobs converting the FHIR clinical data format—which is currently supported directly by vendors of electronic health records into other data storage formats such as OMOP and i2b2—are unnecessary. The largest challenge for the FHIR standard is the ability to use the data for further analysis. Nonetheless, even research-driven formats such as OMOP and i2b2 often need further processing for detailed statistical analysis. For example, for further data analysis using DataSHIELD, a distributed privacy preserving data analysis platform, further processing of OMOP and i2b2 data is necessary [26]. This indicates that direct processing of FHIR resources can reduce the overall complexity and help avoid extra transformation steps.

This study shows that the use of PSQL to store FHIR data and further build web-based preprocessors on this infrastructure is a viable way to handle large amounts of clinical data without having to rely on cloud-based or proprietary data storage solutions. This not only retains a hospital’s ownership of its data but also allows the hospital to avoid vendor lock-in. Development of the preprocessor as a webservice implies that integration into web-based tools can be easily achieved, and the generation of a web-based JavaScript user interface, for example, can be inherently supported. The tool developed in this study does not require the FHIR data to be harmonized across hospitals; however, cross-hospital data analysis is only viable if data are harmonized. Direct integration into the DIC infrastructure developed across Germany and the DIC ensuring data harmonization, including LOINC mapping for laboratory values and LOINC harmonization and unit harmonization through conversion, would facilitate future multicenter studies. Using 3 clinically meaningful scenarios and a real-world data set, we demonstrated the usefulness of the developed framework .

This study integrated the preprocessing service with the KETOS environment and directly interacted with the preprocessor from within a Jupyter Notebook. We made the preprocessor available only within the KETOS platform, allowing it to be password protected by default. Deployment of this platform within a hospital—after pseudonymizing the data and confining it to the hospital for further analysis—ensures patient privacy. Specifically, this framework facilitates retrospective analyses of large data sets, where consent for the data to leave the hospital confines cannot be reasonably obtained. Duplication of this framework across institutions allows data custodians and researchers within each institute to perform analyses and then collaborate with researchers from other institutions. The prerequisite for this is that only aggregated data leave the confines of each institution for the final analysis. In a potential workflow, researchers can establish the preprocessing specifications and analysis scripts with a Jupyter Notebook at their institution and share them with collaborators. This allows them to not only check and execute the scripts at their institution but also modify the scripts per their data requirements, if necessary. Aggregated results or generated models can then be shared across the collaborating institutions. Throughout the process, the FHIR format and identical preprocessing ensures that the scripts and specifications are applicable across the institutions.

It is important to note that the preprocessor only generates SQL queries and does not have large hardware requirements because search and filtering are carried out by the well-established open source PSQL database. A more detailed performance test of the implementation is beyond the scope of this study because performance largely depends on database optimization and indexing, and the number of resources identified for the base filter criteria. However, even the longest requests to generate our sample datasets took minutes rather than hours, despite only creating basic indices for resource types and IDs.

### Lessons Learned

The development of a preprocessor based on FHIR data stored in PSQL jsonb databases for statistical analysis is a viable alternative, facilitating more advanced data processing when compared to the FHIR Search specified as part of the FHIR standard. The FHIR format itself is suitable for querying because JSON queries can be used to specify preprocessing input parameters. The performance of the PSQL database is limited insofar as handling of large data is strongly influenced by how well the PSQL database is administered. For the database we used in this study, we defined some simple indices on the basis of the fhir_id and the resource type to improve the query performance. Here, we first attempted to implement the preprocessor directly on a FHIR server; however, we found that the HAPI FHIR server did not perform well with large bulk loads, which led to the DIC switching from the HAPI FHIR server to the PSQL database. Therefore, large amounts of data were never directly available in a FHIR server. More complex queries, including pattern searches and combining of data for filtering across resources, were not directly supported by the HAPI server. The initial implementation of the preprocessor based on the HAPI server first downloaded the necessary resources to be processed within the preprocessor; however, this was less efficient than direct processing of the data on the database side. The current implementation focuses on feature selection, wherein one particular feature is selected, and inclusion and exclusion criteria are based on the sought-after feature in relation to other data. A cohort selection process could be implemented by selecting the distinct patient IDs in the result set. A future version of this platform should investigate how these concerns could be separated. A feature selection module can then be built on top of a cohort selection module in a 2-step process.

### Generalizability and Use in Other Studies

Reliance on the FHIR format and, more specifically, on fields within the FHIR resources, which are usually set, implies that the proposed method is applicable in various scenarios without requiring further ETL jobs. The preprocessor could process any combination of Observations, Procedures, and Conditions identified by their code within the respective vocabulary. The implementation is currently restricted to the filtering of individual base resources, implying that the generation of data sets where multiple resources are associated with one another based on groups is currently not supported. One could envision an extension, which combines the results of multiple queries into one data set in the future, allowing for more complex analysis. The current version will support the extraction and investigation of any single feature in relation to others. In this study, we demonstrate the investigation of, for example, hemoglobin levels. Any other laboratory value, condition, or procedure would be supported by the current platform. In particular, the method proposed here allows one to filter each occurrence of a feature individually, implying that one query can filter individual occurrences of a feature over time. This facilitates queries, such as the search for hemoglobin value observations around which no blood transfusions have occurred.

### Limitations

The preprocessor specified and implemented in this study was developed on the basis of one projects’ requirement on data handling. Although this study demonstrates its applicability in various scenarios, it does not satisfy more advanced query mechanisms including those developed by , for example, the OMOP OHDSI group. For instance, this framework lacks deeper temporal logic [27], such as temporal filters (eg, the first observation after a certain event). Furthermore, it is important to note that the preprocessor cannot be directly used to define patient cohorts and feasibility queries because it focuses on extracting one feature in relation to others over time. While this restricts the use of the tool, it allows for more specific identification of individual feature occurrences in relation to others. The preprocessor implemented here cannot provide the extent of out-of-the-box analysis which the OMOP and i2b2 tool suites provide; however, it clearly demonstrates the feasibility of building preprocessing tools for FHIR-formatted data. Overall, the data selection and extraction processes specified here have to be used in combination with analysis tools such as DataSHIELD or Jupyter Notebooks, allowing researchers to apply use-case–specific analysis tools to the extracted data or, in the case of DataSHIELD, use the data sets for cross-hospital analysis.

Further, this preprocessing service is dependent on JSON input, and it lacks a user interface. Finally, building on top of a PSQL database restricts the preprocessor to the PSQL database, which implies that some of the interoperability that the FHIR standard aims at is lost in the process, and the current solution cannot replace an FHIR server. However, as the data has to be transformed for analysis regardless, it still provides a viable alternative for FHIR data storage for further analysis.

### Future Directions

This study shows that PSQL jsonb lends itself well to being extended with preprocessing services for data modeling. Further studies are required to investigate how to create a preprocessing tool for the FHIR format, which has similar capabilities to those of the OHDSI OMOP ATLAS or the i2b2 querying tools. In this pursuit, studies should evaluate whether the existing tools already implement all necessary logic for developing and analyzing statistical models. The preprocessor developed here currently lacks a user interface, which is an important requirement for any preprocessor to make it more accessible to a wider audience with different technical backgrounds. We recommend the development of a user interface as an important subsequent step while simultaneously improving this preprocessor. Furthermore, studies should investigate how well different FHIR databases lend themselves to advanced processing of data needed to generate a data set for statistical analysis. For practical reasons (ie, the data being available in our consortium in a simple PSQL database containing one table), we built the preprocessor on top of this PSQL schema. Depending on the outcome of the analysis of the available FHIR stores, a cohort and feature selection mechanism could be developed on the fhirbase project or other solutions, including the clinical quality language capable blaze FHIR store or an extended FHIR search specification and implementation. Criteria-based resource selection is only a small part of a larger analysis framework, similar to OHDSI OMOP and i2b2, which is currently missing for the FHIR standard and should be developed in the future. However, even for larger data sets, direct preprocessing on FHIR resources is a feasible alternative and should be further investigated.

### Conclusion

The preprocessor developed in this study demonstrates how standard open source tools including PSQL can be used to store FHIR data in a format that can be used to generate further filtering, cohort, and feature selection mechanisms. We further deployed the tool at the University Hospital Erlangen-Nürnberg and applied the preprocessor to a large pool of data, generated 3 sample data sets, and executed analyses on top of the generated data sets to demonstrate the applicability of this preprocessor in research. These queries included multiple FHIR resources, such as Observation, Condition, Procedure, Patient, and Encounter, demonstrating the capability of our implementation.

## Appendix

Multimedia Appendix 1 Example query json specification.

Multimedia Appendix 2 Example query generated sql.

## Abbreviations

DIC: data integration center
ETL: extract, transform, load
FHIR: Fast Healthcare Interoperability Resources
i2b2: Informatics for Integrating Biology and the Bedside
JSON: JavaScript Object Notation
jsonb: binary JavaScript Object Notation
MI-I: German Medical Informatics Initiative
PSQL: PostgreSQL
