# Supplementary material for: A Framework for Criteria-Based Selection and Processing of Fast Healthcare Interoperability Resources (FHIR) Data for Statistical Analysis: Design and Implementation Study
Source: JMIR Med Inform. 2021 Apr 1;9(4):e25645. doi: 10.2196/25645 (PMC8050750; doi:10.2196/25645)
Supplement: Multimedia Appendix 1 [file medinform_v9i4e25645_app1.pdf]

MultimediaAppendix 1 – Example query json specification


```
{
  "loinc": ["718-7"],
  "include": [
     {
           "resource": "Observation",
           "system": "http://loinc.org",
           "search_type": "exact",
           "dateField": "effectiveDateTime",
           "codes": ["718-7"],
           "value_restriction":{
              "operator": ">",
              "compare_to": 9
           },
           "time_interval": 2
        }
  ],
  "exclude": [
     {
           "resource": "Observation",
           "system": "http://loinc.org",
           "search_type": "exact",
           "dateField": "effectiveDateTime",
           "codes": ["718-7"],
           "value_restriction":{
              "operator": "<",
              "compare_to": 8
           },
           "time_interval": 0
        }
  ],
  "feature_set": [
     {
        "resource": "Patient",
        "name": "pid",
        "resource_val_path": "identifier.0.value"
     },
     {
        "resource": "Patient",
        "name": "sex",
        "resource_val_path": "gender"
     },
     {
        "resource": "Observation",
        "name": "age",
        "computed":{
           "field_1":{
              "resource": "Observation",
```

```
            "resource_val_path": "effectiveDateTime"
          },
          "field_2":{
            "resource": "Patient",
            "resource_val_path": "birthDate"
          },
          "operation": "diffYearsDate"
        }
      },
      {
        "resource": "Observation",
        "name": "val",
        "resource_val_path": "valueQuantity.value"
      }
  ]
}
```
