# Supplementary material for: A Framework for Criteria-Based Selection and Processing of Fast Healthcare Interoperability Resources (FHIR) Data for Statistical Analysis: Design and Implementation Study
Source: JMIR Med Inform. 2021 Apr 1;9(4):e25645. doi: 10.2196/25645 (PMC8050750; doi:10.2196/25645)
Supplement: Multimedia Appendix 2 [file medinform_v9i4e25645_app2.pdf]

Multimedia Appendix 2 – Example query generated sql

```
WITH base AS
  (SELECT *
   FROM resources
   WHERE TYPE = 'Observation'
     AND resources.data -> 'code' -> 'coding' -> 0 ->> 'code' = '718-7' ),
     enc AS
  (SELECT *
   FROM resources
   WHERE TYPE = 'Encounter'),
     incl AS
  (SELECT *
   FROM resources
   WHERE (TYPE = 'Observation'
          AND DATA -> 'code' -> 'coding' -> 0 ->> 'code' IN('718-7')
          AND TO_NUMBER(DATA->'valueQuantity'->>'value', '99.9') > 9)),
     excl AS
  (SELECT *
   FROM resources
   WHERE (TYPE = 'Observation'
          AND DATA -> 'code' -> 'coding' -> 0 ->> 'code' IN('718-7')
          AND TO_NUMBER(DATA->'valueQuantity'->>'value', '99.9') < 8))
SELECT *
FROM base
WHERE base.fhir_id IN
    (SELECT DISTINCT base.fhir_id
     FROM base
     LEFT JOIN incl ON base.data -> 'subject' ->> 'reference' = incl.data -> 'subject' ->> 'reference'
     LEFT JOIN enc ON incl.data -> 'encounter' ->> 'reference' = concat('Encounter/', enc.data ->> 'id')
     WHERE (((TO_TIMESTAMP(base.data ->> 'effectiveDateTime', 'YYYY-MM-DDTHH24:MI:SS') >=
TO_TIMESTAMP(enc.data -> 'period'->> 'start', 'YYYY-MM-DDTHH24:MI:SS') - interval '2 days'
              AND TO_TIMESTAMP(base.data ->> 'effectiveDateTime', 'YYYY-MM-DDTHH24:MI:SS') <=
TO_TIMESTAMP(enc.data -> 'period'->> 'end', 'YYYY-MM-DDTHH24:MI:SS') + interval '2 days')
             OR (TO_TIMESTAMP(base.data ->> 'effectiveDateTime', 'YYYY-MM-DDTHH24:MI:SS') >=
TO_TIMESTAMP(incl.data -> 'period'->> 'start', 'YYYY-MM-DDTHH24:MI:SS') - interval '2 days'
                 AND TO_TIMESTAMP(base.data ->> 'effectiveDateTime', 'YYYY-MM-DDTHH24:MI:SS') <=
TO_TIMESTAMP(incl.data -> 'period'->> 'end', 'YYYY-MM-DDTHH24:MI:SS') + interval '2 days'))
            AND incl.data -> 'code' -> 'coding' -> 0 ->> 'code' IN('718-7')
```

```
        AND TO_NUMBER(incl.data->'valueQuantity'->>'value', '99.9') > 9) )
AND base.fhir_id NOT IN
  (SELECT DISTINCT base.fhir_id
   FROM base
   LEFT JOIN excl ON base.data -> 'subject' ->> 'reference' = excl.data -> 'subject' ->> 'reference'
   LEFT JOIN enc ON excl.data -> 'encounter' ->> 'reference' = concat('Encounter/', enc.data ->> 'id')
   WHERE (excl.data -> 'code' -> 'coding' -> 0 ->> 'code' IN('718-7')
        AND TO_NUMBER(excl.data->'valueQuantity'->>'value', '99.9') < 8) )
AND TYPE = 'Observation'
AND base.data -> 'code' -> 'coding' -> 0 ->> 'code' = '718-7'
```
